# The hidden diversity of the potato cyst nematode *Globodera pallida* in the south of Peru

**DOI:** 10.1111/eva.12896

**Published:** 2019-12-03

**Authors:** Romain Thevenoux, Laurent Folcher, Magali Esquibet, Didier Fouville, Josselin Montarry, Eric Grenier

**Affiliations:** ^1^ IGEPP INRA Agrocampus Ouest Université de Rennes 1 Le Rheu France; ^2^ Laboratoire de la santé des végétaux ‐ Unité de nématologie ANSES – Agence nationale de sécurité sanitaire de l’alimentation, de l’environnement et du travail Le Rheu France

**Keywords:** genetic differentiation, genetic diversity, microsatellites, speciation

## Abstract

Our knowledge of the diversity of potato cyst nematodes in their native areas still remains patchy and should be improved. A previous study based on 42 Peruvian *Globodera pallida* populations revealed a clear south to north phylogeographic pattern, with five well‐supported clades and maximum diversity observed in the south of Peru. In order to investigate this phylogeographic pattern more closely, we genotyped a larger collection of Peruvian populations using both cathepsin L gene sequence data and a new set of 13 microsatellite loci. Using different genetic analyses (STRUCTURE, DAPC), we consistently obtained the same results that led to similar conclusions: the presence of a larger genetic diversity than previously known suggesting the presence of cryptic species in the south of Peru. These investigations also allowed us to clarify the geographic borders of the previously described *G. pallida* genetic clades and to update our knowledge of the genetic structure of this species in its native area, with the presence of additional clades. A distance‐based redundancy analysis (dbRDA) was also carried to understand whether there was a correlation between the population genetic differentiation and environmental conditions. This analysis showed that genetic distances observed between *G. pallida* populations are explained firstly by geographic distances, but also by climatic and soil conditions. This work could lead to a revision of the taxonomy that may have strong implications for risk assessment and management, especially on a quarantine species.

## 1 INTRODUCTION

Species delimitation is still a challenging area in the field of biology and more precisely in taxonomy and systematic science. Isolated populations accumulate genetic differences across their genomes as they diverge, whereas gene flow between populations counteracts this divergence. Speciation occurs by the accumulation at specific loci of mutations that reduce the fitness of hybrids, thereby preventing gene flow (Abbott et al., 2013; Seehausen et al., 2014). Thanks to genomic data and appropriate modelling, many cases in the so called “grey zone” between species and population have been resolved for animals. These improvements have led to the discovery of numerous cryptic species or species complexes (Bickford et al., 2007; Nygren, 2014; Roux et al., 2016). Cryptic speciation seems to occur mainly in groups characterized by low active dispersal abilities (Casu & Curini‐Galletti, 2003; Neusser, Jorger, & Schrodl, 2011; Roux et al., 2016) including nematodes (Derycke et al., 2008; Palomares‐Rius, Cantalapiedra‐Navarrete, & Castillo, 2014).

Potato cyst nematodes (PCN) are a major pest of potato native to South America. An extensive sampling campaign was carried out in Peruvian potato fields in 2002 in order to improve our understanding of the evolutionary history of *Globodera pallida* (Picard, Plantard, Scurrah, & Mugniery, 2004), one of the two well‐known Andean potato cyst nematode species. However, as we progress towards an understanding of the evolutionary history of this particular species, the general idea that the orogeny of the Andes has triggered a variety of adaptive biotic radiations has become a key notion regarding *Globodera* species evolution and specialization (Grenier, Fournet, Petit, & Anthoine, 2010). At this time, only four *Globodera* species parasitizing potato have been identified. Among them *G. leptonepia* was found only one time in a ship‐borne consignment of potatoes. It is presumed to be a South American species parasitizing potato, but extensive field collections of potato cyst nematodes in the Andean highlands (Evans, Franco, & Descurrah, 1975) have not resulted in its rediscovery. As a result, *G. leptonepia* remains a rare and poorly known PCN. *G. ellingtonae* is a recently described PCN species (Handoo, Carta, Skantar, & Chitwood, 2012). Initially found and described from a potato field sampled in Oregon (USA), this species seems to be restricted geographically to the Americas at this time (Skantar et al., 2011). The last two PCN species are the well‐known *G. pallida* and *G. rostochiensis* species that both originate from the Andes (Grenier et al., 2010).

Cryptic species should not be ignored as they are important for a number of applied reasons regarding in particular food security, risk assessment or nonchemical management technologies. In the case of quarantine nematodes like PCN, failure to recognize cryptic species might complicate efforts towards their eradication or management and also has strong economic consequences for potato export. The question of whether *G. pallida* should be considered a species complex rather than a single species has been raised by several authors (Madani et al., 2010; Subbotin, Prado Vera, Mundo‐Ocampo, & Baldwin, 2011). Interestingly, previous investigations carried out on *G. pallida* populations sampled along the Andean Cordillera in Peru have revealed a phylogeographic pattern from south to north, with five distinct clades (named I–V) (Picard, Sempere, & Plantard, 2007), and high nucleotide divergence (10%–11% based on cytochrome B sequencing) between populations belonging to the southern and northern clades (Picard et al., 2007). This first study on the genetic diversity of *G. pallida* was conducted on a limited population set (44 along a 3000‐km transect (Picard et al., 2007; Plantard et al., 2008)), and made use of a set of seven microsatellite loci available at that time. Since then, novel microsatellite loci have been developed directly from *G. pallida* genome (Cotton et al., 2014), selected and combined together to develop a new robust genotyping tool based on 13 microsatellite loci (Montarry et al., 2019, 2015).

We worked here on a set of 117 PCN populations sampled in the geographic area where European PCN originate (Boucher et al., 2013; Plantard et al., 2008), and where the highest genetic diversity was observed for *G. pallida* (Picard et al., 2007). We used two types of molecular markers. First, populations were genotyped using the intron length polymorphism of the cathepsin L gene. The cathepsin L gene is involved in nematode nutrition and is made up of 12 introns (Blanchard, 2006). Considerable intron length polymorphism has already been reported for several nematode genes among different *Globodera* species (Alenda, Gallot‐Legrand, Fouville, & Grenier, 2013; Geric Stare et al., 2011). The cathepsin L gene is no exception regarding this intron length polymorphism, and in particular, the region spanning introns 4 and 5 were found to be particularly polymorphic among PCN species (Blanchard, 2006). In fact, based solely on the amplification length polymorphism of this cathepsin region, it is possible to distinguish *G. pallida* from *G. rostochiensis* or *G. ellingtonae*. Second, populations were genotyped using the new set of 13 microsatellite markers. Contrary to the cathepsin L gene, microsatellite loci are often species‐specific markers and are located in the noncoding part of the genome (Li, Korol, Fahima, Beiles, & Nevo, 2002; Selkoe & Toonen, 2006).

Thanks to the increase in the number of populations from South Peru investigated and to the use of two different genotyping tools with different tempos of evolution, our objectives were (a) to explore the geographic distribution of *G. pallida*, *G. rostochiensis* or *G. ellingtonae* in South Peru and (b) to reinvestigate more in‐depth the genetic diversity of *G. pallida* in its native area.

## 2 MATERIALS AND METHODS

### 2.1 Cathepsin genotyping

A total of 117 Peruvian and two Chilean populations were studied and are listed in Table S1 along with their geographic location. All are from the laboratory collection and were multiplied on the potato cv “Désirée.” We sampled populations from different multiplication years to avoid any effect of the year of multiplication on our data. In all, eight juveniles at the L2 stage from eight different cysts of each population were pooled, and their DNA was extracted using the NaOH protocol as describe in Boucher et al. (2013) and genotyped using the cathepsin L primers (Forward: 5' AATCKGTRGATTGGCGTGAC 3'; Reverse 5' GGGCCTTGDGTKGCAACAGC 3'). PCR was carried out in a total volume of 25 µl (12.25 µl of ultra‐purified H_2_O, 5 µl of 5 × buffer, 3 µl of MgCl_2_ at 25 mM, 1 µl of each primer at 10 µM, 0.25 µl of Taq Golfex 5 U/µl, 0.5 µl of dNTPs at 10 µl each and 2 µl of DNA). The PCR conditions were a denaturation step at 95°C for 1 min, followed by 30 cycles (denaturation: 95°C for 30 s, annealing: 63°C for 50 s, elongation: 72°C for 1 min) and a final elongation step at 72°C for 5 min. Amplification products were observed after migration on an agarose gel (1.5%). The amplification products obtained for European *G. rostochiensis* populations are about 705 bp, amplification products for European *G. pallida* populations are about 690 bp, and those for *G. ellingtonae* are about 880 bp (Figure S1).

### 2.2 Microsatellite genotyping

*Globodera pallida* populations studied with microsatellites were selected based on the results obtained following cathepsin L genotyping. Where a mix of species was observed in one population, we excluded *G. rostochiensis* individuals when there was a majority of *G. pallida*. Not all populations had sufficient individuals to be genotyped by this tool, and finally, 84 populations were studied and none had less than 24 individuals; the precise number of individuals retained per population for each analysis is specified in Table S1.

The genotyping protocol, including the loci and primers used, was the same as in Montarry et al. (2015, 2019). Briefly, for each population, 40 cysts were randomly collected and one juvenile (L2 stage) sampled from each cyst. DNA extraction was performed as described in Boucher et al. (2013). All individuals were genotyped at 13 microsatellite loci: Gp106, Gp108, Gp109, Gp111, Gp112, Gp116, Gp117, Gp118, Gp122, Gp126, Gp135, Gp145 and Gr67, and genotyping was performed on the Gentyane INRA platform. Allele sizes were identified using the automatic calling and binning procedure of Genemapper v 5.0 (Do & Rahm, 2004) and completed by a manual examination of irregular results.

We used two different data sets for analysis. In the first data set, we chose to retain the maximum number of populations and then removed loci that did not amplify enough individuals in each population. This first data set consisted of 84 populations genotyped by 10 microsatellite loci (loci Gp112, Gp135 and Gp145 were excluded) and was named “10locix84pop.” In the second data set used for further analysis, we chose to retain all the genetic information (all 13 loci) and then removed populations where no or too few individual amplifications were obtained (populations TDF, CAS, 309, 384, 264 and 224 were excluded). This second data set was named “13locix78pop.”

### 2.3 Population genetic descriptors and genetic structure analysis

Different population descriptors like the observed and expected heterozygosity, the fixation index (*F*_IS_ (Wright, 1978)), and the allele diversity, were calculated using Poppr package (Kamvar, Tabima, & Grünwald, 2014) in R and using the rarefaction index (ElMousadik & Petit, 1996).

To observe the genetic differentiation between populations, we calculated pairwise *F*_ST_ (Weir & Cockerham, 1996) using HIERFSTAT (Goudet, 2005) in R software (R Development Core Team 2018), which is based on allelic frequencies. Genetic structure was also investigated using a Bayesian model based on the Markov chain Monte Carlo (MCMC) clustering method and implemented using the STRUCTURE program, v 2.2.3 (Pritchard, Stephens, & Donnelly, 2000). We performed five independent runs; each had 1,000,000 cycles of burn‐in and 3,000,000 cycles of MCMC, with the number of clusters tested (*K*) ranking from 2 to 50. This analysis assumes an admixture ancestry model based on allele frequency. Results were analysed with pophelper (Francis, 2017), implemented in R. To assess the optimal values of *K*, we used the Δ*K* method described by Evanno, Regnaut, & Goudet (2005), as well as a visual comparison between the different *K* values tested. To supplement the output from STRUCTURE, we also analysed the data sets trough a discriminant analysis of principal components (DAPC) (Jombart, Devillard, & Balloux, 2010), using the ADEGENET package for R (Jombart, 2008). To assess the optimal values of *K*, we used the Bayesian information criterion (Bickford et al., 2007). We checked the stability of individual group membership probabilities and the correct number of principal components (PCs) using the α‐score (Jombart et al., 2010) and cross‐verification.

### 2.4 Phylogenetic analysis

To investigate the relationship between populations, we conducted a phylogenetic analysis using Poppr (Kamvar et al., 2014) and phytools (Revell, 2012) in the R package. We used Nei dissimilarity (Nei, 1978) and built trees with the unweighted pair group method with arithmetic mean (UPGMA) (Sokal & Michener, 1958). We calculated a node statistical support, running 999 bootstraps of resampling loci.

### 2.5 Geographic distances and the impact of climatic and soil conditions

We tested the isolation‐by‐distances (IBD) hypothesis following instruction of Rousset (1997) and using the Vegan package (Oksanen, Blanchet, Kindt, Legendre, & O'Hara, 2018). The statistical significance of the correlation between the genetic distances (*F*_ST_/(1 − *F*_ST_)) (Slatkin, 1995) and the natural logarithm of geographic distances were estimated with a Mantel test (10,000 permutations) using Vegan package (Oksanen et al., 2018).

We also conducted a distance‐based redundancy analysis (dbRDA; Legendre & Anderson, 1999) to correlate *F*_ST_ values with abiotic and climatic variables. We chose six variables which, from our knowledge, could impact the distribution of PCN. These variables could be geographic (latitude and longitude), climatic (mean annual temperature, mean annual precipitation) and pedologic (content in organic carbon, cation exchange capacity), or they could reflect plant diversity (Shannon index). These variables were extracted from WorldClim v2 (Hijmans, Cameron, Parra, Jones, & Jarvis, 2005), SoilGrid (Hengl et al., 2017) and EarthEnv (http://www.earthenv.org) based on population GPS coordinates. We performed dbRDA using the Capscale function implemented in the Vegan package (Oksanen et al., 2018) in R, and we finally chose the best model using a stepwise model based on an adjusted R2 and *p*‐value (Ordistep function, Vegan package) from a start saturated model including all variables cited above, and latitude and longitude, to take into account the geographic position:

(1) $$\begin{array}{c} F_{\text{ST}}∼\text{Annual mean temperature}+\text{Annual mean precipitation}+\text{Organic carbon}+\text{Cation exchange}\\ +\text{Water capacity}+\text{Shannon Index}+\text{Latitude}+\text{Longitude} \end{array}$$

## 3 RESULTS

### 3.1 Cathepsin analysis results

Amplification of the cathepsin marker on 119 populations revealed either one or two bands of different sizes. Overall, four amplification products of different sizes were observed with a length of approximately 690, 705, 800 and 845 bp (Table S1). Amplification products of 705 bp are known to correspond to *G. rostochiensis*, while amplification products of 690 bp correspond to *G. pallida*. None of the investigated populations appeared to correspond to *G. ellingtonae*, but amplification products of unexpected sizes (i.e. 800 bp and 845 bp) were observed. The 800 bp product was observed in 10 populations from the northern part of the studied area, and always in association with the fragment corresponding to *G. pallida*, except in population 196. The 845 bp product was observed in 22 populations often in association with the fragment corresponding to *G. pallida*, but in four cases (224, 264, 284 and 309) as a unique amplification product. In all, 25 populations were uniquely or mostly constituted by *G. rostochiensis* (705 bp amplification product), and we removed them from further investigations because of the difficulty in obtaining sufficient *G. pallida* individuals in the mixed populations. All *G. rostochiensis* individuals found were located in the south of the study area.

In PCN populations, maximum diversity was observed in the south of our study area, around Lake Titicaca, where three out of the four sizes of amplification products were found. The 800 bp profile was mostly found in the north of our study area, and usually in association with the 690 bp amplification corresponding to *G. pallida*. Only one population (196) was found with solely the 800 bp amplification product. This 800 bp product size was subsequently further observed in other Peruvian populations from clades IV and V (Table S1). The 690 bp product was the most commonly observed in our data set and is also found in association with all the other amplification products in mixed populations. Since the PCRs were conducted on a pool of eight juveniles, we cannot determine whether the two amplification products observed in some populations appeared because of hybrids or because of the heterogeneity of the pool. On the contrary, the 845 and 800 bp amplification products were each shared by less than 16% of the populations. Using additional PCN populations from South America, we were able to identify the 845 bp amplification product in two PCN populations sampled in the South of Chile, in Patagonia (TDF and CAS). Finally, we noted a clear geographic distribution of the cathepsin L gene diversity, with the 800 bp amplification product located in the north. We then found mostly the 690 bp allele when going to the south, where additional amplification products can be observed (i.e. 705 and 845 bp).

### 3.2 Microsatellite missing data and genetic differentiation

Among all populations, few missing data were observed except for populations 309, 284 and 264, which were not amplified by the loci Gp112, Gp135 and Gp145. 60% of individuals from population 224 were amplified by these loci. Importantly, these populations were those showing the 845 bp cathepsin L amplification product. We also examined the two populations from south Chile (TDF and CAS) and found that they also did not amplify these three loci. These Chilean populations were therefore included in some of the subsequent analyses, and the group formed by populations TDF, CAS, 309, 284, 264 and 224 will now be referred to as the “pallida Chilean type.”

*F*_ST_ values are distributed from 0 to 0.5697 (Figure S2). A clear separation into five groups was observed, with lower *F*_ST_ values between populations belonging to the same group than between populations belonging to different groups. Values > 0.5 are observed between populations belonging to the “pallida Chilean type” and the other populations.

### 3.3 Genetic structure

#### 3.3.1 Observed structuration using the “10locix84pop” data set

**Figure 1:**
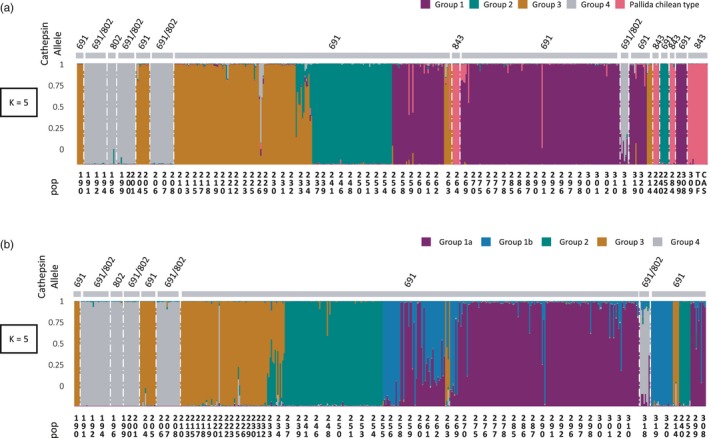
STRUCTURE clustering analysis obtained with the two different data sets. (a) Results obtained with the “10locix84pop” data set where three loci were removed and all populations were taken into account (b) Results obtained with the “13locix78pop” data sets where all loci were taken into account and “pallida Chilean type” populations were removed; the y‐axis shows the assignation rate of each individual displayed on the x‐axis. White vertical lines show the transition between different cathepsin alleles indicated on the top of each STRUCTURE graph

**Figure 2:**
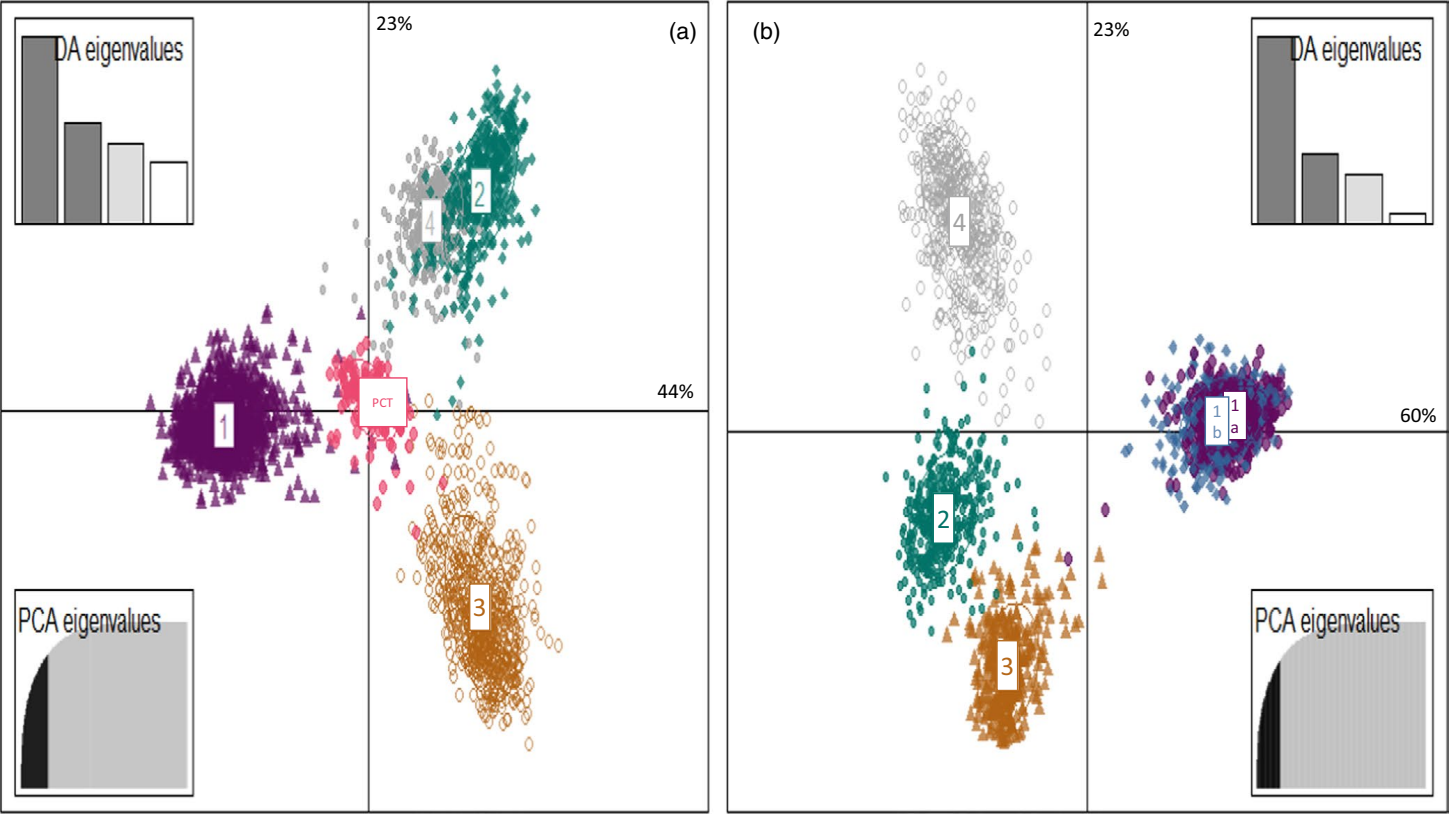
DAPC analysis obtained with the two different data sets. (a) Scatterplot showing the dispersion of individual of the DAPC analysis for data set “10locix84pop” where three loci are removed and all population taking in count. (b) Scatterplot showing the dispersion of individual of the DAPC analysis for data set “13locix78pop” where all loci are taking in count and “pallida Chilean type” population is removed. Percentage of Da eigenvalues and number of PCA axis retained for each analysis are present of the side of each graph

The best model in STRUCURE separated genetic variation into five clusters (Figure 1a), with a clearly highest Δ*K* for *K* = 700 (Figure S3). Once again, populations from the south of Chile were grouped with populations 309, 284, 264 and 224. The exact same structuration into five groups was observed with both STRUCTURE (Figure 1a) and DAPC (Figure 2a). We also found perfect congruence with the cathepsin L genotyping results. Interestingly, when there was a switch of the cathepsin allele, there was also a switch of group determined by the STRUCTURE analysis (Figure 1). Overall, individuals and populations were both clearly assigned to only one group, except for populations 233 and 234, which were assigned to both group 2 and group 3.

#### 3.3.2 Observed structuration using the “13locix78pop” data set

**Figure 3:**
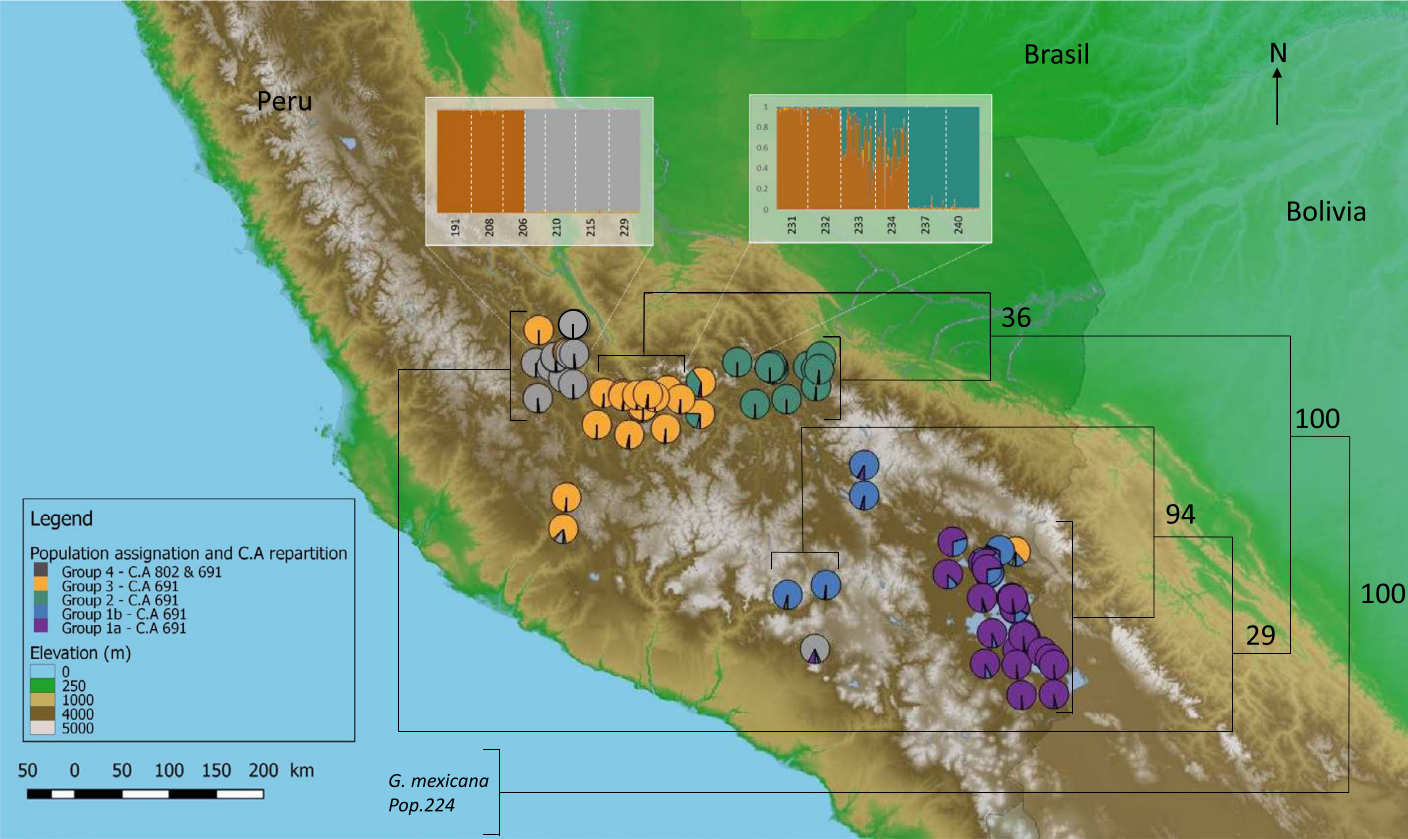
Map presenting structuration results. Pie charts with indicated colours represent the mean assignation of the populations at each group. A consensus UPGMA dendrogram using Nei distances is also presented; bootstrap values are presented at each node. Two foci of the STRUCTURE results at the individual scale are presented for 2 × 6 populations located at admixture geographic areas. The Cathepsin Alleles (C.A) are indicated in the legend according to the genetic groups allowing a representation of their geographic distribution

This data set of 78 populations, all from Peru and including all loci, showed an optimal structuration at *K* = 5 (Figures 1b, and S4). The results from DAPC were similar and supported clustering into five groups (Figure 2b). We could expect structuration into four groups due to the removal of the “pallida Chilean type” populations, but populations 320, 319, 255 and 256 split from group 5 to form a new group. The structuration of this data set is clearly linked to the geographic position of populations (Figure 3), with mostly longitudinal differentiation between groups 1a and 1b. An assignation of each population to each group is provided in the Table S1. The descriptive genetic variables do not show differences between groups (Table S1), except for the allelic richness, which is lower for group 3.

Some particularly interesting cases are observed from STRUCTURE outputs. Most individuals from populations 233 and 234 are assigned half to group 2 and half to group 3 (Figure 3), indicating some admixture between these two groups. Considering geographical data, these populations are located exactly at the edge of these two groups on a plateau delimited by two valleys. For comparison, another case is provided in Figure 3 with populations 191, 208, 206, 210, 215 and 229 all located in the same geographic area. In this case, we did not observe any admixture between groups 2 and 4. We also observed that there are major geographic barriers between all groups, except between groups 1a and 1b (Figure S5).

### 3.4 Phylogenetic approach

We chose to incorporate as the out‐group for this analysis, individuals from *G. mexicana*, the closest relative to *G. pallida*, and individuals from population 224 belonging to the “pallida Chilean type.” After generating trees from different genetic distances, we chose to present a UPGMA dendrogram built with Nei distances, the most supported tree (Figure 3). Population 224 seems to be as different from the other Peruvian populations as *G. mexicana*. A high bootstrap value supports this result, as well as the first phylogenetic partition between branches corresponding to groups 1a, 1b and 4 on one side, and 2 and 3 on the other. Bootstrap values are also high for the divergence between groups 1a and 1b. The other nodes are less well supported; nevertheless, we can note that the groups identified by the phylogenetic analysis match those identified by STRUCTURE or DAPC analyses.

### 3.5 Geographic distances and the impact of climatic and soil conditions

**Figure 4:**
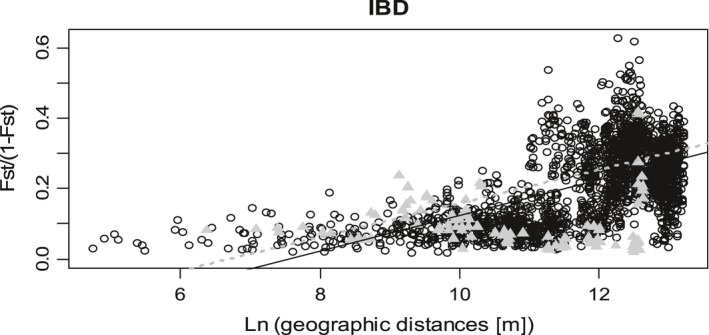
Isolation‐by‐distance pattern between genetic differentiation and geographic distances for pairwise populations. Genetic differentiation is expressed as (*F*_ST_/(1 − *F*_ST_)) and geographic distance as natural logarithm of the distance in metres. Populations from group 4 are in grey triangle. Dashed grey line stands for correlation when group 4 is excluded and dark line for correlation when all populations are included

We found a highly significant IBD pattern when considering either, all populations or all populations without the ones from group 4 (Figure 4). A significant correlation was observed between genetic and geographic distances considering all populations (Mantel test, Pearson's coefficient of correlation = 0.41, *p* = .000001). This correlation is even higher when we exclude from the analysis populations from group 4 (Mantel test, Pearson's coefficient of correlation = 0.59, *p* = .000001).

**Figure 5:**
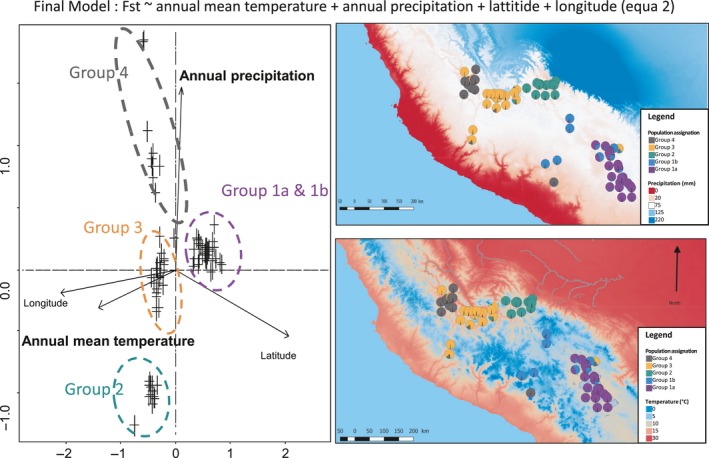
Influence of climatic variables on the genetic differentiation of *G. pallida* populations. (a) Plot of dbDRA results, from the final model (Equation 2), crosses stand for populations, and coloured circles indicate the groups. (b) Map of the annual mean precipitation in the study area between 1970 and 2000. (c) Map of the annual mean temperature between 1970 and 2000. On these maps, populations and their assignation to each group are plotted

From the saturated model (Equation 1), the best and final model (equa 2, see Fig. 5) retained the annual mean temperature and precipitation variables, as well as the latitude and the longitude.

## 4 DISCUSSION

The first aim of this study was to explore more precisely the genetic diversity within *G. pallida* in the cradle of the species. We found, as previously shown, a clear genetic structure in this area, linked to the geographic but also pedoclimatic conditions. We identified two groups (i.e. the “pallida Chilean type” and group 4) that may be cryptic species within the *G. pallida* species complex. Furthermore, we recorded the presence of *G. rostochiensis* in the studied area and the absence of *G. ellingtonae*. All populations of *G. rostochiensis* were found in the south of Peru, which is consistent with previous observations (Evans et al., 1975; Franco, 1977). The absence of *G. ellingtonae* in our data set is in line with the possible origin of this species outside Peru (Lax, Duenas, Franco‐Ponce, Gardenal, & Doucet, 2014).

### 4.1 Alignment with previous results

The microsatellite genotyping results showed that our populations are structured in a maximum of six groups (1a, 1b, 2, 3, 4 and “pallida Chilean type”), which are geographically distributed from south to north Peru. In parallel, the cathepsin L marker identified three groups, the first corresponding to groups 1a, 1b, 2 and 3, the second to group 4, and the third to the “pallida Chilean type.” Compared with the conclusions reached by Picard et al. (2007), groups 1b and 4 were detected for the first time here. Our groups 1a and 1b correspond in fact to clade I from Picard et al. (2007). However, only one population belonging to group 1b (i.e. pop 320) was included in the data set used by Picard et al. (2007). Located northward, group 2 corresponds to clade II, and group 3 to clade III described in Picard et al. (2007). The “pallida Chilean type” was detected by all analyses and with two types of markers; however, it was not identified by Picard et al. (2007) who did not consider any of these populations in their study. Similarly, Subbotin et al. (2011) carried out a phylogenetic analysis of South American populations of *G. pallida* using ITS‐rRNA sequences. Subclade 1 in Subbotin et al. (2011) seems to correspond to groups 1a and 1b identified here. They also suggested that three of their subclades may represent putative undescribed species. First, subclade 3 that contains populations also studied here and that was identified as belonging to the “pallida Chilean type.” Second, subclade 5 which contains northern Peruvian populations that were not studied here, but that are known to show the same cathepsin L amplification product as that seen in group 4. Third, subclade 6 composed of two populations, one of which is population 235 belonging here to group 2.

Overall, our results are still consistent with those reported by Picard et al. (2007) and Subbotin et al. (2011). The three additional groups observed here can be attributed to the use of an extended and improved set of microsatellite markers, and to the higher number of populations sampled. The use of next‐generation sequencing tools could be helpful to investigate genetic variation at a larger number of loci and covering the entire genome (Davey et al., 2011). Despite the location and delimitation of groups highlighted here are consistent with previous results, our phylogenetic outputs are in contradiction with them. The hypothesis of evolution linked to Andes orogenesis proposed by Picard et al. (2007) implies northward differentiation; consequently, group 4 should diverge from group 3. Results from our analyses show that southern populations (groups 1a and 1b) and northern populations (group 4) are phylogenetically more closely related than group 4 is related to groups 2 and 3. However, the phylogenetic approach followed by Subbotin et al. (2011) showed a different story where northern populations and populations from group 2 diverged earlier from the “pallida Chilean type” and southern populations. Our results rather place the “pallida Chilean type” as an out‐group compared with the other *G. pallida* populations studied. However, we should be cautious when interpreting the results of our phylogenetic analysis because of the low confidence associated with nodes lower than 0.5.

### 4.2 A possible differentiation pattern

With the exception of the ambiguous delimitation between groups 1a and 1b where gene flow still occurs, the other groups were well separated by all the analyses conducted here. A very important point is the consistency of results from the two different genetic markers used that follow different evolution models. A second point is the consistency between outputs from the STRUCTURE and DAPC analyses which have different assumptions. This distinction between markers and analyses could even provide us with insights into the range of differentiation between the observed groups.

Groups 1, 2 and 3 are readily detected by all analyses, but share the same cathepsin allele, which demonstrates their genetic proximity (Geric Stare et al., 2011). They should therefore be considered populations of the same species which explains why hybrids (pop. 233 and 234) are observed between groups 2 and 3 (Coyne & Orr, 2004). The proportion of hybrid individuals in these two populations reaches 60% and suggests that individuals are viable and fertile. Clearly, if they were not, and if hybrid depletion was in place, the hybrid proportion in the population would be lower than the proportion of individuals in each group. As a result, the hypothesis of viable and fertile hybrids is retained here. Comparatively, we observed no similar assignation between groups 3 and 4, while they are geographically close. Only very few individuals can be considered hybrids. This could be due to hybrid depletion and lead to high differentiation between these two groups, comparable to the distinction between *G. pallida* and *G. rostochiensis*, species for which hybrids are observed but are not fertile (Mugniéry, Bossis, & Pierre, 1992). Furthermore, group 4 presents a unique cathepsin amplification product of 800 bp. This leads us to suspect that group 4 is more genetically differentiated than the others. This view is also supported by the IBD patterns observed as the correlation coefficient is lower when population from group 4 is included in the analysis. This suggests that group 4 did not follow exactly the same evolution compared with the other groups. This hypothesis is also supported by the outputs from the phylogenetic analysis.

The question now is to determine whether this differentiation observed here has engendered speciation. Genetic studies are helpful when considering cryptic species, but it is too speculative to conclude about species delimitation without information from other fields (Padial, Miralles, Riva, & Vences, 2010; Wheeler, 2004; Will & Rubinoff, 2004). For example, crossing test has already provided clarification for the *Globodera* genus (Mugniéry et al., 1992) and could be carried out in this case.

### 4.3 Climatic and soil conditions potentially explain genetic differentiation

Beside the impact of geographic distance and relief, our results also showed the importance of climatic conditions in the genetic differentiation observed. The dbRDA analysis separated mostly group 4 from group 2 by precipitation conditions, and group 1 from the others by temperature. Group 4 evolved in drier conditions, while group 1 is located in a more temperate zone around Lake Titicaca. We could imagine that these particular conditions lead to some genomic adaptations, and thereby to genetic differentiation. We note that the variable plant diversity seems to have no effect on the differentiation between groups. This finding should be considered with caution, because in parasite species, hosts are known to be a driver of speciation (de Vienne et al., 2013). The variable chosen here to reflect host plant diversity (Shannon index) could appear to be approximative because it is built to reflect habitat heterogeneity but not intra‐taxon heterogeneity, such as in *Solanaceae*, which is the host of *G. pallida*. However, we could imagine that the climatic conditions (AMT and AMP) affect the diversity and distribution of *Solanaceae* and thereby the differentiation of *G. pallida*. Moreover, the available climatic and soil data used here are from the last 30 years, while the differentiation is probably older. Therefore, we have to hypothesize that different climatic conditions were at least conserved or even higher at the time the genetic differentiation of these groups occurred.

### 4.4 The “pallida Chilean type” as a candidate for a new species

Populations forming the “pallida Chilean type” were clearly identified by both DAPC and STRUCTURE analysis. This group also appears to be the most genetically distant in our data set, and several results support the idea that the divergence is as high as that between different species. First, *F*_ST_ showed that this group is the most distant from the others. *F*_ST_ values calculated between *G. pallida* and “pallida Chilean type” populations always reached values> 0.5, which are similar in magnitude to the *F_ST_* values observed between Peruvian *G. pallida* and *G. rostochiensis* populations (i.e. 0.58, data not shown) or between *G. pallida* and *G. mexicana* (i.e. 0.48, data not shown). Second, it should be noted that three microsatellite loci failed to amplify most of, if not all, individuals belonging to this group. This type of failure of amplification is also observed for *G. rostochiensis* individuals using the Gp111, Gp112 and Gp145 loci (data not shown). Third, the phylogenetic analysis suggests that population 224, which belongs to the “pallida Chilean type,” is not phylogenetically related to the *G. pallida* species history, and rather clusters outside *G. pallida*, like the out‐group species *G. mexicana,* used to root the tree. Nonetheless, the question remains open, as other results are conflicting. The recent molecular tools developed for quarantine PCN diagnosis (Gamel, Letort, Fouville, Folcher, & Grenier, 2017) are not able to distinguish these “pallida Chilean type” populations from the other *G. pallida* populations, while they are able to distinguish *G. mexicana* from *G. pallida*.

Geographically, the “pallida Chilean type” populations are found in the south of our study area, in sympatry with populations belonging to groups 1a and 1b. One surprising result is that this “pallida Chilean type” has never been identified in Europe despite the fact that (a) both *G. rostochiensis* and *G. pallida* were introduced from the same geographic area (Boucher et al., 2013; Plantard et al., 2008), and (b) the cathepsin genotyping results showed that the “pallida Chilean type” is found even more often in sympatry with *G. pallida* (17 out of 26 co‐occurrences) than *G. rostochiensis* is found in sympatry with *G. pallida* (9 out of 26 co‐occurrences). It could therefore be hypothesized that this group was in fact also introduced into Europe, but failed to establish itself and survive in European conditions. This once again suggests strong differences with *G. pallida* also in terms of fitness and life‐history traits that would rather support the view of different species. Clearly, further investigations are required before being able to conclude on a novel PCN species. In a perspective of integrative taxonomy (Dayrat, 2005; Padial et al., 2010), the morphologic and behavioural fields should now also be explored.

The evolutionary history of the “pallida Chilean type” remains mostly unknown. At this time, populations belonging to this group have only been reported in Chile (Patagonia) and south Peru. The genetic proximity observed between these populations that are distant by more than 6,000 km is quite surprising, considering the divergence observed for *G. pallida* in Peru. The best explanation is therefore that—in contrast to *G. pallida* phylogeographic history mostly due to Andes orogenesis—the “pallida Chilean type” phylogeographic history is rather the result of anthropic dispersion between these two countries. Considering the low levels of heterozygosity and allelic richness observed in the CAS and TDF populations, compared with the 224, 264, 284 and 309 populations, it can even be assumed that these populations were introduced to Chile from south Peru. These two countries were at war until 1884 (end of the War of the Pacific), with battles that were fought in the Pacific Ocean, but also mostly in the Atacama Desert and mountainous regions of the Peruvian and Bolivian Andes. Similarly to what occurred for the PCN species *G. rostochiensis*—detected in the United States for the first time in 1942 in Long Island and probably introduced with contaminated military equipment returning from Europe after the First World War (Brodie & Mai, 1989)—it is possible that the War of the Pacific was the event that allowed for such long‐distance dispersion.

The possible presence of a species complex may be important in terms of economic impact on crop harvests and must be taken into account if a quarantine species is involved. This is particularly true for *G. pallida* which is listed as a quarantine species because of the significant damage that it could produce in potato crops. A description of the potential new species would imply revision of its taxonomy and expand *G. pallida *sensu* stricto* to *G. pallida *sensu* lato*. The groups identified as potential new species appear to be absent from Europe, but they could be introduced. The current regulations and controls on *G. pallida*, that is, the ban on introduction into Europe of soils originating from non‐EU countries, are able to prevent this risk of new introduction. Nonetheless, reinforced controls applied to at‐risk regions and maintaining *G. pallida* on the list of quarantine nematode species appear important, if not essential.

## CONFLICT OF INTEREST

The authors have no competing interests to declare.

## Data Availability

The microsatellite genotyping data sets are available at Data Inra (https://data.inra.fr) under the https://doi.org/10.15454/IZ2JSN (Thevenoux, Folcher, Esquibet, Fouville, & Grenier, 2019).
