# Supplementary material for: The hidden diversity of the potato cyst nematode Globodera pallida in the south of Peru
Source: Evol Appl. 2019 Dec 3;13(4):727–37. doi: 10.1111/eva.12896 (PMC7086051; doi:10.1111/eva.12896)
Supplement: Supplementary file 1 [file EVA-13-727-s001.pdf]

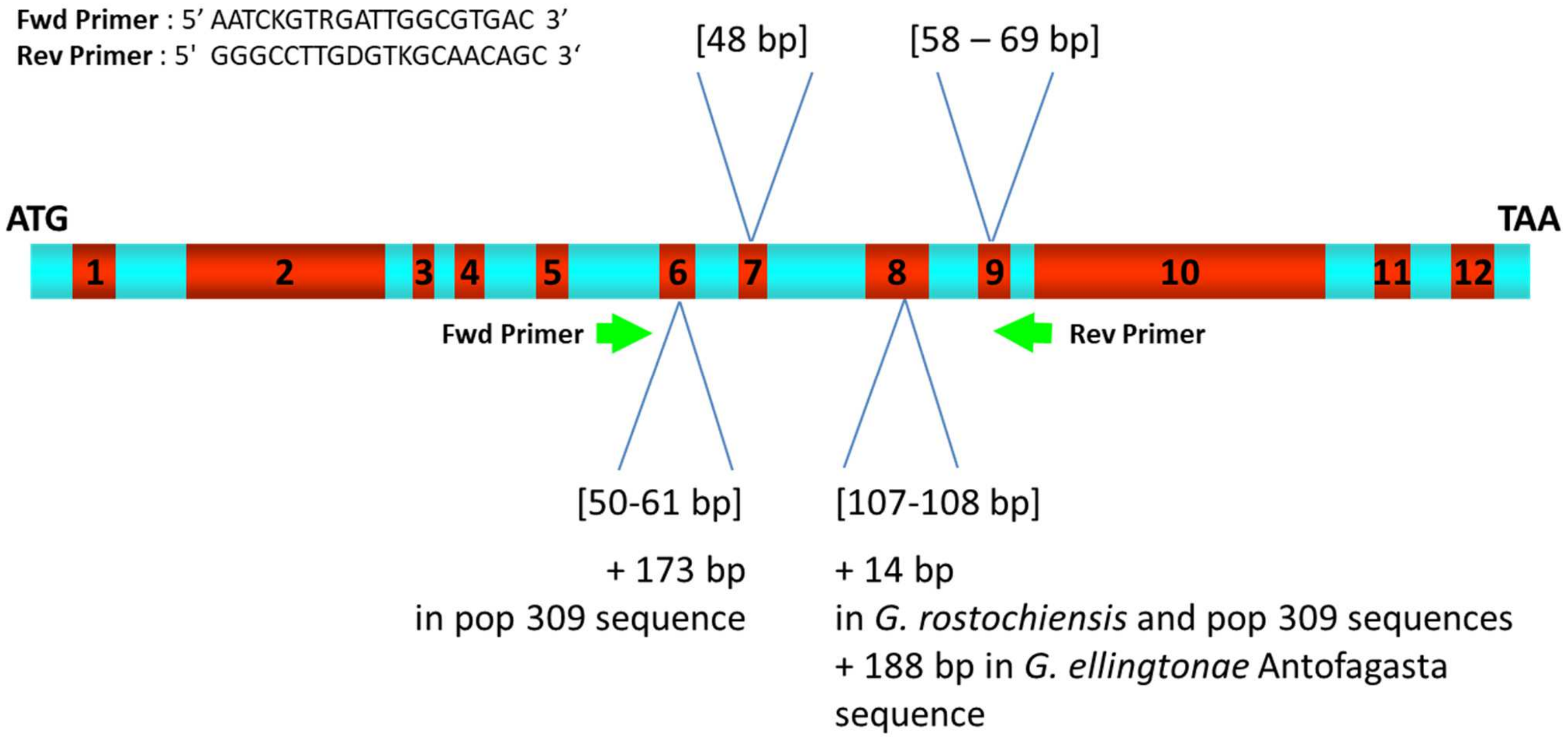

Fwd Primer : 5’ AATCKGTRGATTGGCGTGAC 3’
Rev Primer : 5' GGGCCTTGDGTKGCAACAGC 3‘
[48 bp]
[58 – 69 bp]
ATG
TAA
1
2
3
4
5
6
7
8
9
10
11
12
Fwd Primer
Rev Primer
[50-61 bp]
+ 173 bp
in pop 309 sequence
[107-108 bp]
+ 14 bp
in G. rostochiensis and pop 309 sequences
+ 188 bp in G. ellingtonae Antofagasta
sequence

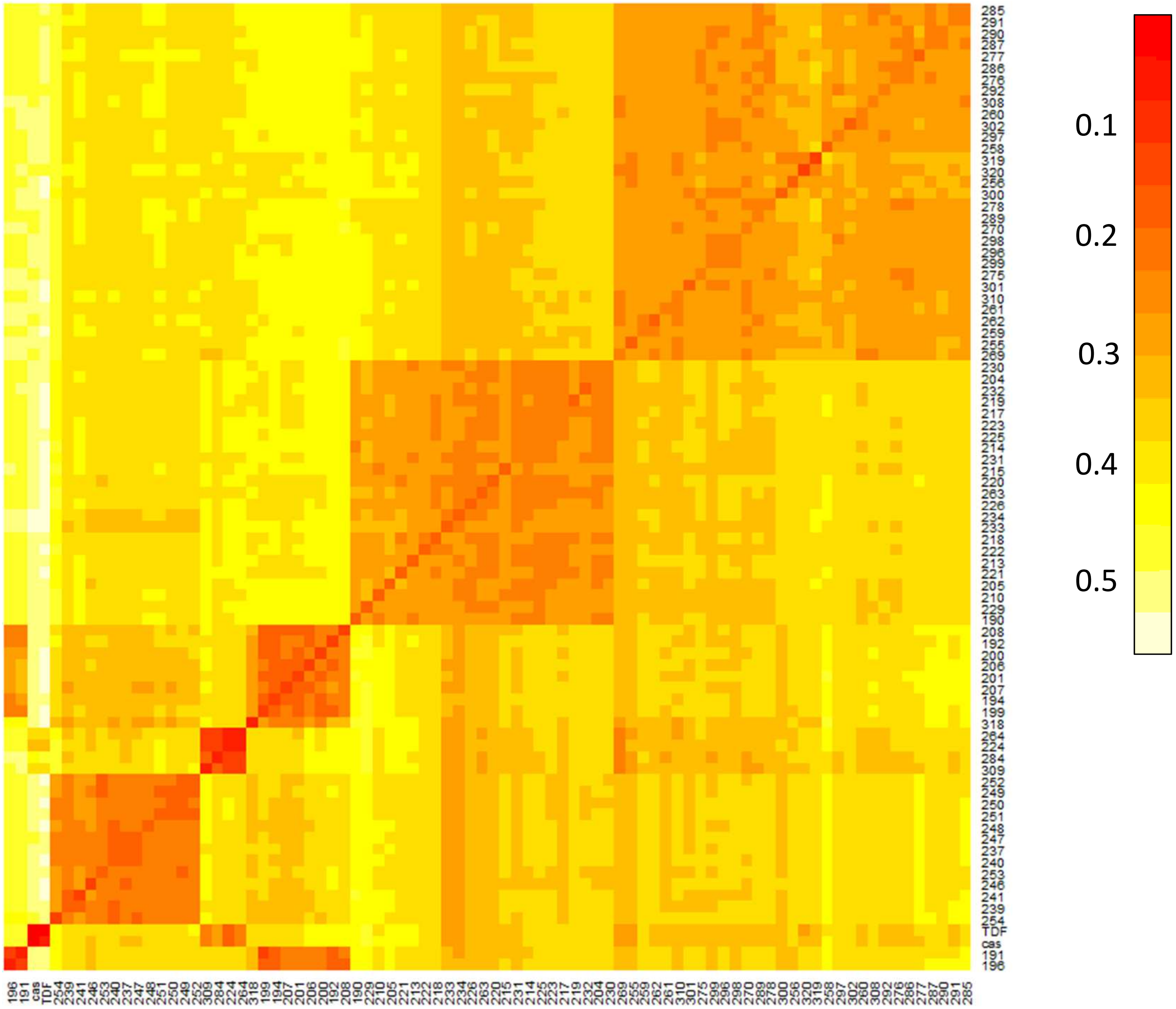
0.1
0.2
0.3
0.4
0.5
285
291
290
287
277
286
276
292
308
260
302
297
258
319
320
256
300
278
289
270
298
296
299
275
301
310
261
262
259
255
269
230
204
232
219
217
223
225
214
231
215
220
263
226
234
233
218
222
213
221
205
210
229
190
208
192
200
206
201
207
194
199
318
264
224
284
309
252
249
250
251
248
247
237
240
253
246
241
239
254
TDF
cas
191
196

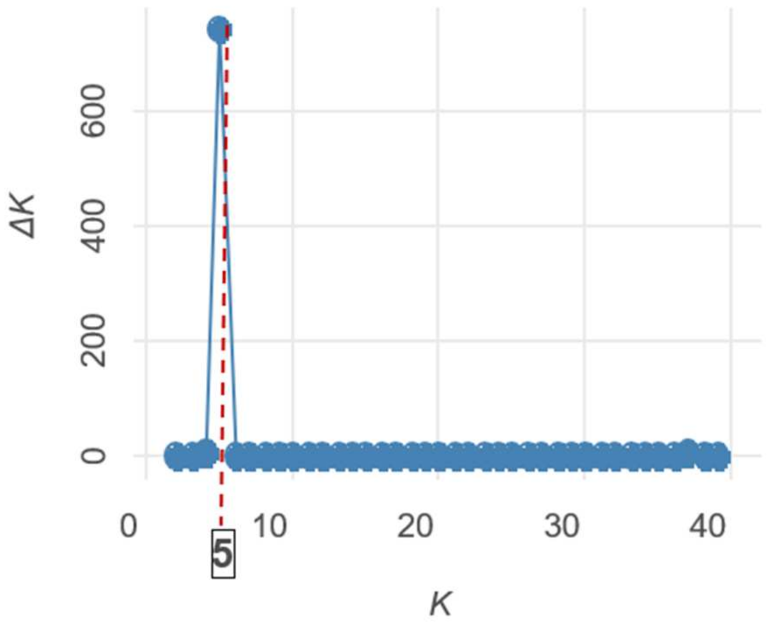

2. STRUCTURE verification for 10 markers

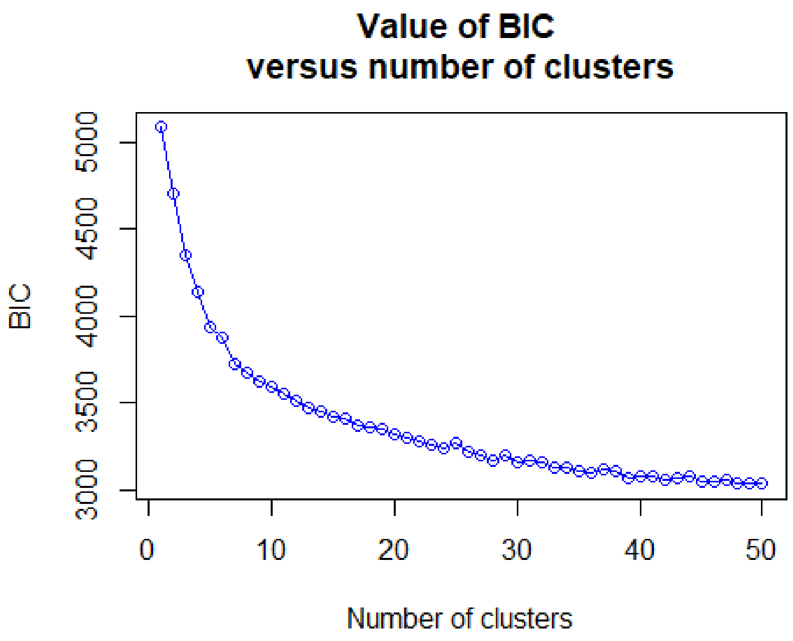

3. Dapc verification for 10 markers

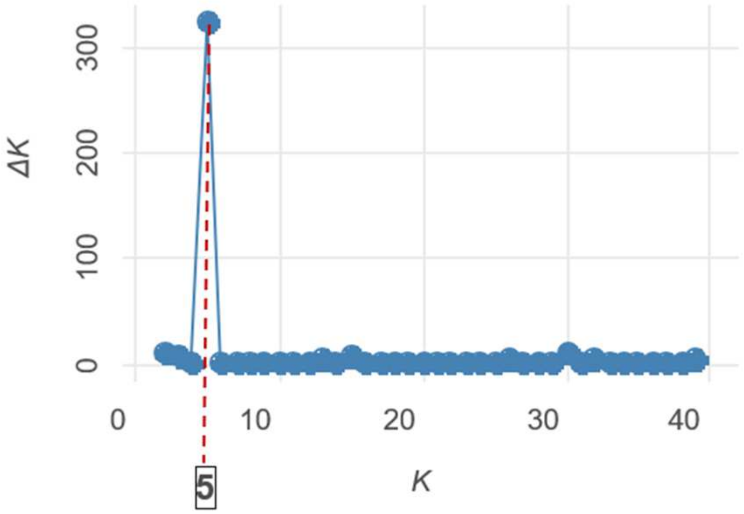

2. STRUCTURE verification for 13 markers

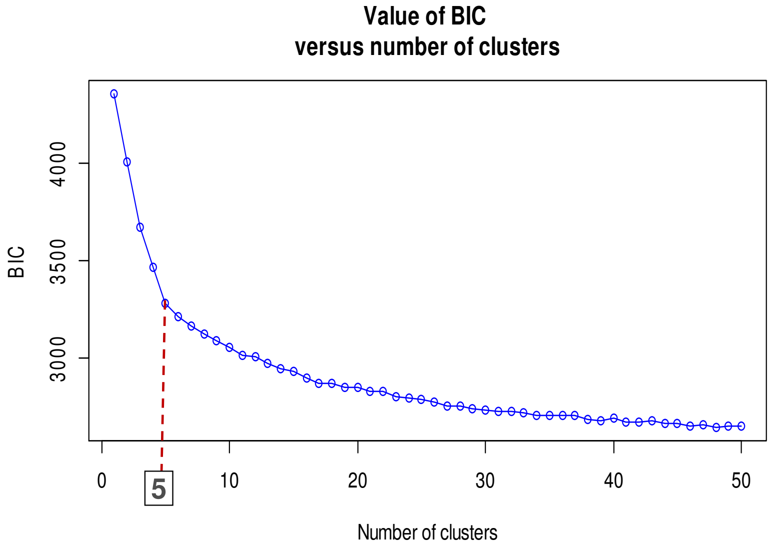

3. Dapc verification for 13 markers

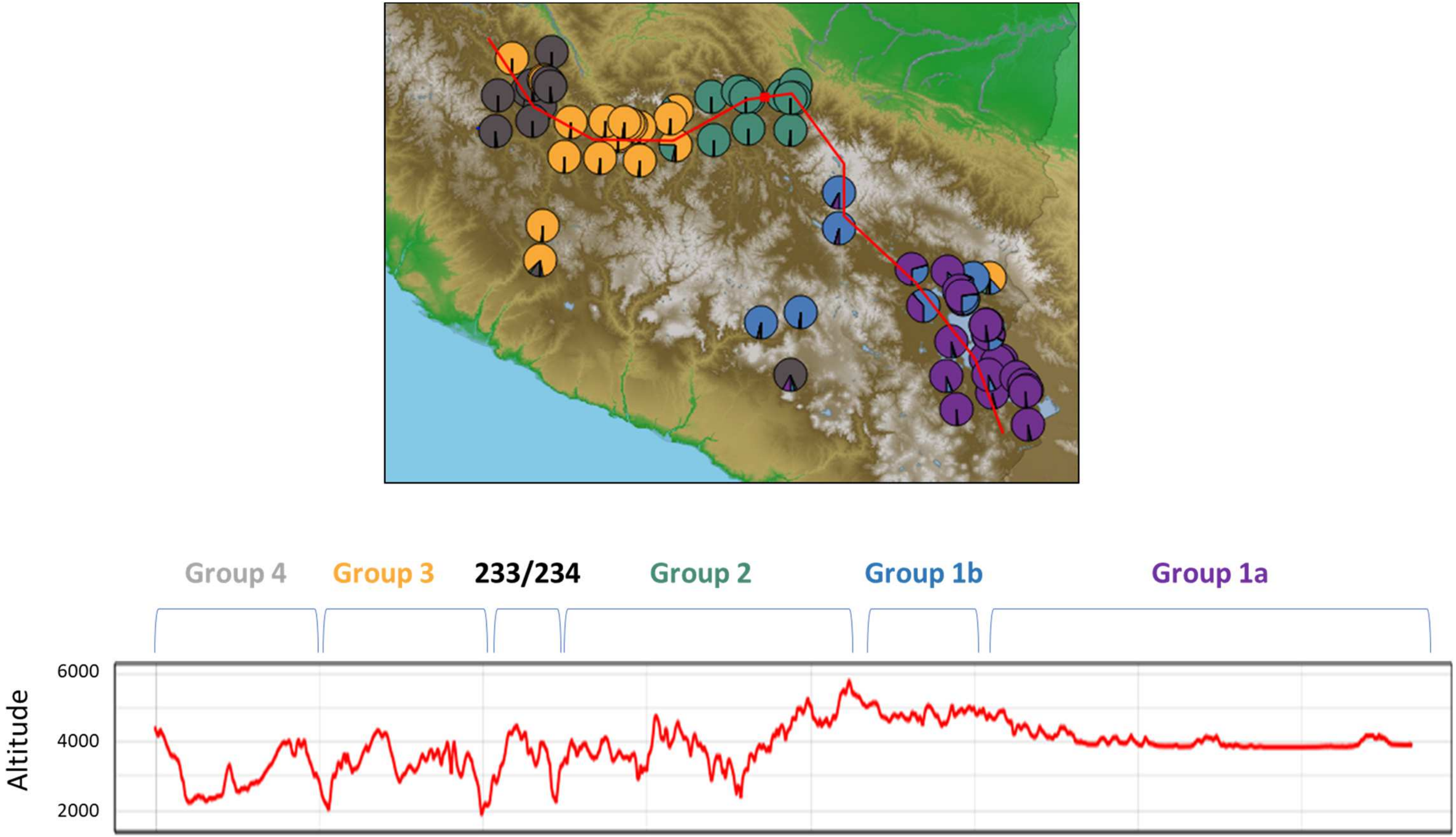
Group 4
Group 3
233/234
Group 2
Group 1b
Group 1a
Altitude
6000
4000
2000
